# Editorial: Datasets for brain-computer interface applications, volume II

**DOI:** 10.3389/fnins.2025.1569216

**Published:** 2025-03-10

**Authors:** Ian Daly, Ana Matran-Fernandez, Mikhail A. Lebedev, Andrea Kübler, Davide Valeriani

**Affiliations:** ^1^Brain-Computer Interfacing and Neural Engineering Group, Computer Science and Electronic Engineering, University of Essex, Colchester, United Kingdom; ^2^National Research University Higher School of Economics, Moscow, Russia; ^3^Department of Psychology I, Institute of Psychology, University of Würzburg, Würzburg, Germany; ^4^Technogym U.K. Limited, Bracknell, United Kingdom

**Keywords:** Brain computer interface (BCI), EEG, ERP, steady-state visual evoked potential (SSVEP), data

Non-invasive Brain-computer interfaces (BCIs) are an exciting technology that provides a channel for communication between the brain and computers. BCIs can be used for communication (Brumberg et al., 2018; Chaudhary et al., 2016), rehabilitation (Cervera et al., 2018), entertainment devices (Gürkök et al., 2017), and a wide range of other applications (Finke et al., 2009; Makeig et al., 2011).

In our first volume of this Research Topic (Daly et al., 2021), we published datasets comprising signals recorded via a wide variety of modalities and BCI paradigms, including novel event-related potential (ERP) and steady state visual evoked potential (SSVEP) based BCIs for communication, motor imagery BCIs, affective BCIs, collaborative BCIs, and neurofeedback-based BCIs for nicotine addiction, as well as resting-state data.

However, research in BCI is continuously developing and there is a growing need for new publicly available datasets. Indeed, continuing development of BCI technology relies on advances made in many different research fields, which individually and collectively can contribute to improving all aspects of BCI systems including signal acquisition, processing, classification, and user interface design.

Despite this, there remains only a small number of high-quality, publicly-available datasets on which new systems, tools, and technologies can be developed, evaluated, and compared. Furthermore, the relatively small size and number of these datasets introduce the risk of overfitting to methods developed and evaluated with these datasets. In other words, the reliability and reproducibility of BCI research may be held back by a lack and sparsity of publicly available datasets.

To continue addressing this challenge, this Research Topic provides a second collection of publications and corresponding datasets. They report on physiological datasets recorded during development, training, and evaluation of non-invasive BCI systems from BCI research labs around the world. Data were collected with electroencephalography (EEG) and functional near infrared spectroscopy (fNIRS). Stimulus presentation within diverse experimental paradigms cover different sensory modalities.

The article by Botrel et al. describes a study on the effects of time and visualization techniques within a neurofeedback paradigm on alpha downregulation and sense of presence in virtual reality. Twenty-five participants were trained for several sessions in two different setups. While subjects learned to control their parietal alpha, no effects on the sense of presence were observed (Botrel et al.).

Functional near infrared spectroscopy is used in the article by Ning et al., who describe data recorded during viewing of complex audio-visual stimuli. A group of 16 adults saw videos of complex natural scenes presented simultaneously on three monitors. Participants were cued to attend to one of the three videos and an initial decoding approach showed above chance level accuracy in determining the participants attentional focus during the tasks (Ning et al.).

Two articles of our Research Topic involve event-related potentials (ERP). In the first study by Reichert et al., a toolbox for decoding ERP-based BCI commands is presented. The toolbox uses canonical correlation analysis and is evaluated on four publicly available BCI datasets (Reichert et al.).

The second study by Lee et al. presents a new dataset recorded from a large cohort of 84 participants who were attempting to use an ERP-based BCI to control a variety of home appliances. Data were collected in a variety of different environments, including the use of LCD display technology to present BCI interfaces, augmented reality, and home environments; significant control was achieved in most cases (Lee et al.).

Finally, a article by Chailloux Peguero et al. presents a dataset recorded during use of an SSVEP-based BCI by a cohort of 27 participants. Different stimuli modulations were used and decoding performances were compared across modulation methods. The results showed that modulating stimuli in a rectangular or sinusoidal on-off pattern and decoding with filter band canonical correlation analysis produces the highest decoding accuracy (Chailloux Peguero et al.).

We hope this second volume of openly available datasets will enable further novel developments and applications of BCI technology, as well as extensive validation studies of current and future BCIs.
